# Metabolic Responses of *Candida viswanathii* on Lipase Production: Influence of Carbon Sources and Culture System

**DOI:** 10.1021/acsomega.5c13021

**Published:** 2026-05-06

**Authors:** Nayra M. L. de Oliveira, Fabrício C. Paula-Elias, Jonas Contiero, Rafaela R. Pinto, Michelle C. A. Xavier, Nayara B. Carvalho, Paula C. Leite, Rafael F. Perna, Sergio A. V. Morales, Alex F. Almeida

**Affiliations:** † Graduate Program in Food Science and Technology, Federal University of Tocantins, Palmas 77001-090, Brazil; ‡ Institute for Research in Bioenergy, São Paulo State University (Unesp), Rio Claro 13506-900, Brazil; § Graduate Program in Chemical Engineering, Institute of Science and Technology, Federal University of Alfenas, Poços de Caldas 37715-400, Brazil

## Abstract

Microbial lipases are pivotal biocatalysts in the food, pharmaceutical, and bioenergy industries due to their versatility and stability. Among potential producers, the yeast *Candida viswanathii* has shown promise, yet the transition from laboratory shake flasks to stirred tank bioreactors often presents challenges due to altered physiological and metabolic responses. Specifically, determining how different carbon sources influence the trade-off between biomass growth and enzyme secretion during this scale-up remains a critical bottleneck. Here we show that while sorbitol acts synergistically with soybean oil to maximize lipase production in shake flasks (13.56 U mL^–1^), it triggers a strong inhibitory effect on enzyme expression when scaled up to a stirred tank bioreactor. Contrary to the expectation that optimized flask conditions would translate linearly, the bioreactor environment shifted the yeast’s metabolism toward rapid biomass accumulation (30 g L^–1^) at the expense of lipolytic activity, with the highest reactor yields achieved only in the absence of sorbitol (13.56 U mL^–1^). These findings underscore the necessity of re-evaluating medium composition specifically for dynamic reactor environments, rather than relying solely on static screening data to predict industrial performance. It is concluded that cultivation in bioreactors for enzyme production must carefully consider agitation and aeration conditions, as these conditions modify the yeast’s behavior, altering nutrient utilization and, consequently, the induction of enzymatic expression and cell growth.

## Graphical abstract

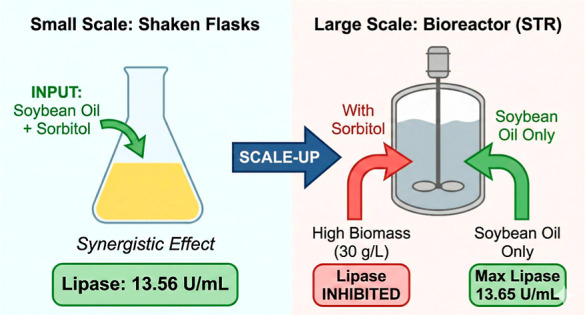

## 1 Introduction

Enzymes are biological catalysts that accelerate chemical reactions and have long been used to improve the shelf life and quality of foods and beverages. In the food industry, microbial enzymes are especially valued due to their higher stability, cost-effective production by fermentation, and ease of optimization compared to plant and animal sources. Increasing demand for enzyme-modified dairy products and concerns related to animal health have further expanded the lipase market.^1,2^ The global microbial lipase market reached USD 643.63 million in 2025 and is projected to grow from USD 682.96 million in 2026 to USD 1118.46 million by 2034, exhibiting a CAGR of 6.36%.^3^

Lipases (E.C. 3.1.1.3) are serine hydrolase enzymes that catalyze the hydrolysis of ester bonds in triacylglycerols, releasing free fatty acids, diacylglycerols, monoacylglycerols, and glycerol.^4^ They preferentially act on long-chain fatty acids (>C10) but are also capable of hydrolyzing short- and medium-chain substrates.^5^ These enzymes are widely applied in food industries for antioxidant synthesis and aromatic ester production,^6–8^ as well as in medicine and pharmaceuticals for diagnostics, antiobesity drugs, and potential cancer treatments.

Microbial lipases, produced by fungi, yeasts, and bacteria, are preferred due to their large-scale fermentation potential, simple nutritional requirements, and high stability across wide pH and temperature ranges. Production occurs via submerged (SmC) or solid-state cultivation (SSC), however, with SmC is highly efficient due to its precise physicochemical control, which can enhance enzyme yields.^9–12^ The improvement in production and subsequent industrial application of lipases must undergo studies in order to establish better cultivation conditions for the microorganisms, and thus to ensure an exponential increase in production.^4,13^ The one-factor-at-a-time (OFAT) optimization approach involves modifying a single variable while keeping all other variables fixed. However, this strategy presents several limitations: it is often labor-intensive and time-consuming, may fail to identify the true optimal conditions, and does not account for potential interactions among variables, since the remaining factors are arbitrarily held constant during the evaluation process.^14^ Statistical models used for optimizing cultivation conditions and increasing lipase production are of paramount importance and have been applied in microbial cultivation. Statistical approaches such as response surface methodology (RSM) and Plackett–Burman designs allow for the structured screening of influential factors and the evaluation of their interactions, thereby contributing to substantial enhancements in process yield.^15,16^ For this purpose, the statistical approach is often practicable, since it allows the analysis of different independent variables, such as carbon and nitrogen sources, inducing sources, physicochemical parameters, among other factors, aiming at one or more response variables. The search for optimal conditions for the production process avoids excessive expenses with random experiments and increases the reliability of the results.^17^

Stirred tank bioreactors are attractive for lipase production because they allow mechanical agitation, medium homogenization and oxygen and nutrient availability, thus favoring the growth of the microorganism and increasing product yield. Aerobic microorganisms require good oxygen transfer, which is related to aeration and agitation rates, to achieve good growth and subsequent enzyme production.^18^ The scaling up of the fermentation process aims to design and build a larger scale system based on the results obtained in bench scale devices.^19^

The yeast *Candida viswanathii* has been extensively studied regarding its high lipolytic potential and characterization, with emphasis on obtaining acid lipase of biotechnological interest. Screening and optimization of submerged cultivation conditions, evaluating different carbon and nitrogen sources, vegetable oils (including amazonian oils), and physicochemical variables that influence enzymatic synthesis demonstrated the potential of *C. viswanathii* for use on an industrial scale.^20–22^ Complementary work involved the analysis of intracellular lipid accumulation associated with lipase production and strategies to increase productivity, and initial investigations of cloning and gene expression of the enzyme consolidate the *C. viswanathii* strain as a promising platform for lipase production with potential application in the food, oleochemical, and biotechnological industries.^23–25^ However, the *C. viswanathii* strain has not been studied in bioreactors in previous works, and this is the first report of lipase production in a mechanically stirred reactor. So, in this work, the production of a lipase by *C. viswanathii* was conducted on a bench scale, evaluating the effect of lipid and nonlipid carbon sources and nitrogen sources, using a rotational central composite design (RCCD). Enzyme production was also verified in a stirred tank bioreactor with and without sorbitol supplementation.

## 2 Materials and Methods

### 2.1 Chemicals

Potato dextrose agar was purchased from HiMedia, Indy. Tryptone, yeast extract and peptone were purchased from Kasvi, Brazil. Sorbitol, Triton X-100, 4-nitrophenol, *p*-nitrophenyl palmitate (*p*-NPP) were purchased from Sigma-Aldrich, EUA. Antifoam Adecanol LG-109 was puchased from Asahi Denka Co., Ltd. Dimethyl sulfoxide and sodium tetraborate were purchased from Dinâmica, Brazil. Residual glycerol was kindly donated by Granol, Brazil. Whey was kindly donated by Puro Leite, Brazil. Vegetable oils were purchased forma local market. All other reagents used in the work were of analytical grade.

### 2.2 Microorganism, Culture Conditions and Reagents

*C. viswanathii* strain is available in the Culture Collection of the Laboratory of Food and Products Analysis, Federal University of Tocantins, Brazil. *C. viswanathii* was cultivated on potato dextrose agar (PDA-slant) for 48 h at 28 °C ± 2 °C, for inoculum preparation. The inoculum was prepared with the cell suspension in a 0.85% NaCl solution, adjusted to 10^7^ cells/mL, using a Neubauer chamber. One milliliter of the suspension was used as inoculum for the submerged cultures. All reagents used in this work were purchased from Sigma-Aldrich and are of analytical grade.

### 2.3 Submerged Cultivation

#### 2.3.1 Selection of the Carbon and Nitrogen Sources

The submerged cultures for the selection of the carbon and nitrogen sources were conducted in Erlenmeyer flasks (125 mL) containing 20 mL of the adapted culture medium described by Dalmau et al.:^26^ dibasic potassium phosphate, 5.5 g·L^–1^; potassium dihydrogen phosphate, 15 g·L^–1^ and magnesium sulfate, 0.5 g·L^–1^. All media were supplemented with 1% (w/v) of olive oil. To test the nitrogen sources, separately in each Erlenmeyer flask, 0.5% (w/v) of corncin, yeast extract, peptone or tryptone were added. To test the influence of the nonlipid carbon source, each medium was supplemented with 0.5% (w/v) of sorbitol, residual glycerol and whey plus 0.5% of yeast extract. All cultures were maintained under orbital shake at 180 rpm and 28 °C ± 2 °C for 36 h. To observe the behavior of lipase production in the medium without supplementation of the carbon and nitrogen sources, a control experiment was prepared, containing only the adapted salts from Dalmau et al.^26^ and 1% of olive oil.

#### 2.3.2 Selection of the Vegetable Oil

The submerged cultures for the selection of vegetable oil sources were performed in Erlenmeyer flasks (125 mL) containing 20 mL of the adapted culture medium described by Dalmau et al.^26^ All media were supplemented with 1% (m/v) of olive oil. To test the vegetable oil sources, separately in each Erlenmeyer flask, 1% of soybean oil, palm oil, pequi oil and sunflower oil were added. Sorbitol, residual glycerol and yeast extract were also added to each flask (0.5%, w/v). All cultures were maintained under orbital shake at 180 rpm and 28 °C ± 2 °C for 36 h.

#### 2.3.3 Optimization of the Submerged Cultivation

Cultivation optimization was performed using the Rotational Central Composite Design (RCCD). For this design, the following variables were used: carbon, nitrogen and vegetable oil sources. RCCD was performed with 17 trials, three of them on the central point at a 5% significance level. The real and coded values are described in Table 1. The data for the factors were chosen after a series of preliminary assays.

**Table 1:** Selected Variables and Their Levels Assigned by RDDC^a^

independent variable	code	–1.68	–1	0	1	+1.68
vegetable oil source	*X*_1_	6.6	10	15	20	23.4
carbon source	*X*_2_	1.6	5	10	15	18.4
nitrogen source	*X*_3_	3.3	5	7.5	10	11.7

a Note: values expressed in g/L.

The analysis of variance (ANOVA) was presented for the quadratic model applied for the enzymatic activity of the lipase from *C. viswanathii*. The adjustment of the experimental responses to the statistical model was evaluated by the coefficient of determination (*R* ^2^) and the *F*-test.

#### 2.3.4 RDDC Validation

To confirm whether RDDC was able to predict enzyme production, an assay was performed with three replications under the best conditions indicated by the model. The predicted and the observed experimental values were compared to verify the efficiency of the proposed experimental design.

### 2.4 Cultivation in the Stirred Tank Bioreactor

Lipase production by *C. viswanathii* was evaluated in a 0.5 L bioreactor (Multifors HT, Boettmingen, Switzerland), from discontinuous cultivation. Cultures in potato dextrose agar were used for the preparation of the preinoculum at 28 °C ± 2 °C for 72 h. A cell suspension in a 0.85% (m/v) NaCl solution was obtained, equivalent to 10^7^ cells mL^–1^.

A volume of 2.5 mL of this suspension was used to inoculate the Erlenmeyer flasks (125 mL) containing 50 mL of salt culture medium, adapted from Dalmau et al.,^26^ supplemented with 23.4 g·L^–1^ of soybean oil (vegetable source) and 7.5 g·L^–1^ of yeast extract (nitrogen source). The cultivation was performed at 180 rpm and 28 °C for 24 h. A volume of 50 mL of the later culture was used to inoculate 450 mL of the salt culture medium, in a stirred tank bioreactor, containing 23.4 g·L^–1^ of soybean oil and 7.5 g·L^–1^ of yeast extract. The experiments without and with sorbitol (10 g·L^–1^) in the medium were verified.

The cultivations in the bioreactor were performed at 28 °C, with the pH maintained at 6 by the automatic addition of 1 M of sodium hydroxide or 1 M of sulfuric acid. The concentration of dissolved oxygen was maintained at 20% of saturated air, under agitation between 100 and 600 rpm and aeration flow between 0.4 and 2.0 vvm. Antifoam Adecanol LG-109 (defoaming agent, Asahi Denka Co., Ltd.) 0.05% was added to the culture medium, when necessary.

### 2.5 Obtaining the Crude Extract

Enzyme extract was obtained after cultivation. For this, using a centrifuge, the crude extract obtained was centrifuged at 10,000*g* for 15 min at 4 °C. The supernatant was used to quantify the lipase activity, and the pellet formed was used to measure the total biomass of the culture.

### 2.6 Determination of the Enzymatic Activity

Lipase activity was determined as described by Almeida et al.^27^ using *p*-nitrophenyl palmitate (*p*-NPP) (Sigma-Aldrich) as substrate. *p*-NPP was initially dissolved in 0.5 mL of dimethyl sulfoxide (DMSO), then diluted to 0.05 M with McIlvaine buffer pH 4.0 containing 0.5% (w/v) Triton X-100. The hydrolysis of pNPP was determined discontinuously by measuring the released *p*-nitrophenol (pNP) at 40 °C. After 5 min preincubation of 0.9 mL of the substrate solution in a water bath, the reaction was started by adding 0.1 mL of appropriately diluted enzyme solution. The reaction was stopped after 1 and 2 min by heat shock (90 °C, 1 min), followed by the addition of 1 mL of saturated sodium tetraborate solution. The absorbance was measured at 405 nm and the activity was determined according to a *p*-nitrophenolate standard curve (ε = 1.8 × 10^4^ M^–1^ cm^–1^). Controls were prepared without enzymes. One enzyme unit (U) was defined as the amount of enzyme that releases 1 μmol of product per mL per min.

### 2.7 Determination of the Biomass

Biomass was estimated after obtaining the crude extract. The pellet formed was washed with distilled water and centrifuged at 10,000*g* for 10 min, and subsequently dried in conical flasks at 60 °C until constant weight.

### 2.8 Determination of the Residual Oil

Four milliliters of the culture supernatant were transferred to centrifuge tubes (15 mL), followed by the addition of 2 mL of hexane. The tubes were vigorously shaken and maintained still until the separation of the organic and aqueous phases. The organic phase was separated and transferred to new tubes, then dried in an oven at 60 °C until constant weight.

### 2.9 Analysis of the Results

The results of the selection of the carbon, nitrogen and vegetable oils sources were analyzed using the software Sisvar version 5.6, using the Scott Knott test at a 95% confidence (*p* ≤ 0.05). The elaboration of the experimental design and statistical analysis of the data was conducted using the software Protimiza Experimental Design.^28^ All assays were performed in three replicates, with analysis in triplicate.

## 3 Results and Discussion

### 3.1 Selection of the Carbon and Nitrogen Sources

Lipase production is strongly related to several factors, such as the types and concentrations of nutrients and the presence, absence and concentration of inducers.^29^ Lipase production by *Candida viswnanathii* was evaluated by the addition of different nitrogen and nonlipid carbon sources to the medium (Figure 1A). In this experiment, cultivations were carried out with olive oil as a sole carbon source. Among the nitrogen sources evaluated, yeast extract showed the highest enzyme production (7.70 ± 0.05 U mL^–1^) followed by tryptone (5.00 ± 0.02 U mL^–1^) and corncin (4.3 ± 0.04 U mL^–1^). Peptone showed the lowest lipase production (0.6 ± 0.00 U mL^–1^). Addition of nonlipid carbon as a sole carbon sources do not induce enzyme production; but among the nonlipid carbon sources plus olive oil, the highest lipase production was observed in the medium supplemented with sorbitol (13.66 ± 0.32 U mL^–1^) followed by glycerol (6.10 ± 0.02 U mL^–1^) and whey (5.80 ± 0.02 U mL^–1^). For the control medium supplemented with only 1% of olive oil without nitrogen sources, no lipase production was observed. The microbial growth under these experimental conditions (Figure 1b) shows that the yeast extract as a nitrogen source presented also the best results (57.5 ± 3.53 g·L^–1^). For the carbon sources, the use of residual glycerol in the culture medium induced the 79.5 ± 0.7 g·L^–1^ of dry biomass, followed by whey (67.25 ± 3.89 g·L^–1^) and sorbitol (61.25 ± 5.30 g·L^–1^). All cultivation media differed significantly (95% confidence interval) from each other for lipase production. For biomass production, the use of corncin and tryptone did not present significant differences while the other carbon and nitrogen sources showed statistical differences among them.

**Figure 1:**
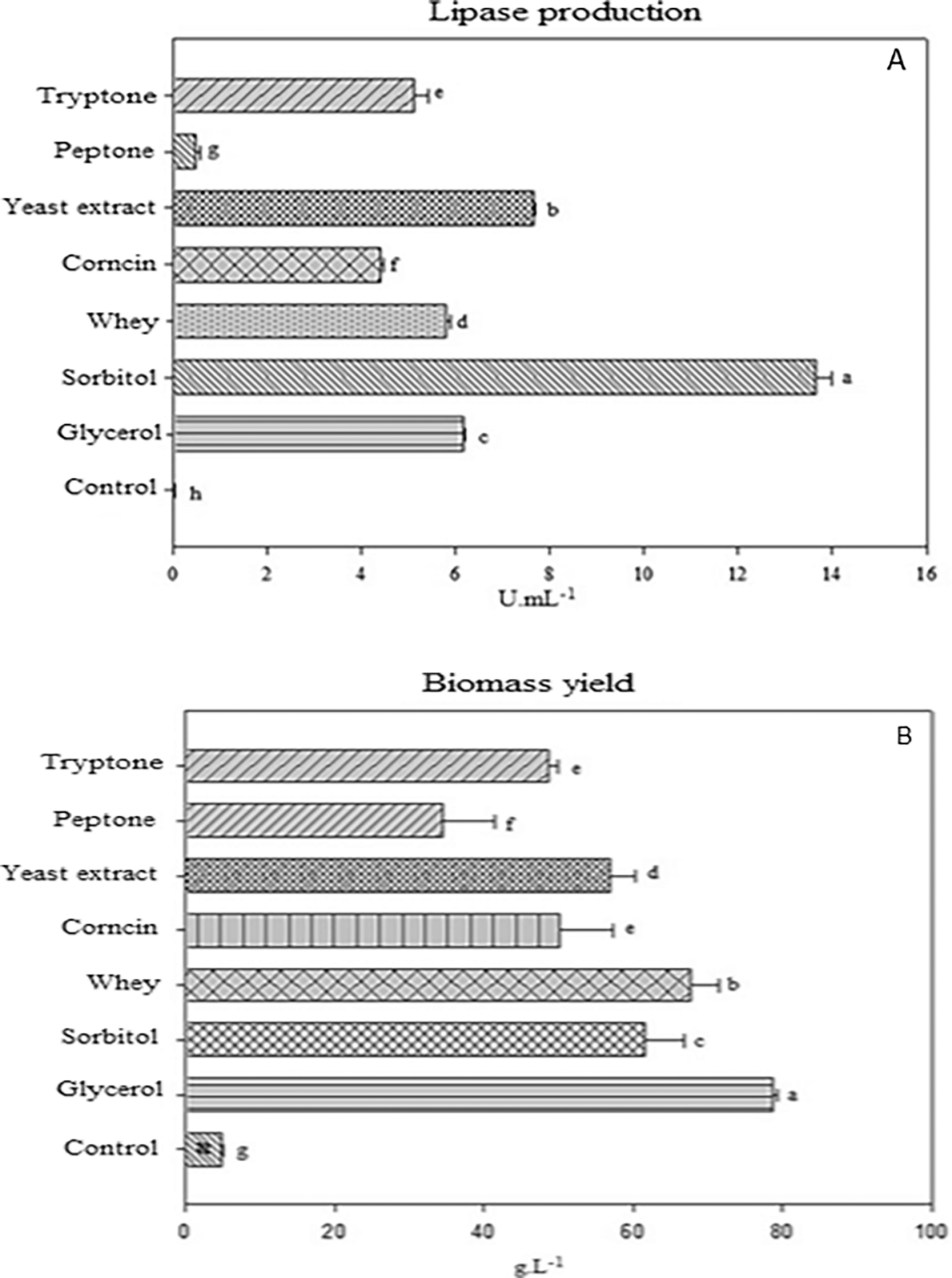
(A) Lipase production (U mL^–1^) and (B) biomass production (g·L^–1^) using different carbon and nitrogen sources. Distinct lowercase letters mean there are significant differences among the media tested at a 95% confidence interval.

Fabiszewska et al.^30^ cultivated *Yarrowia lipolytica* for lipase production. The authors used crude glycerin as a substrate and were able to increase biomass yield in all cultures with yeast growth, but this substrate was not efficient in prolonging the duration of the log phase to increase enzyme production. With high biomass production and lower enzymatic activity, it is evident that glycerol, as a product of triglyceride hydrolysis, may have inhibited lipase production by *C. viswanathii*, even increasing cell growth. Thorpe et al.^31^ used a discontinuous fermentation system for the cultivation of *Pichia pastoris* and verified that the use of glycerol favored the increase in yeast biomass, while the use of sorbitol improved the production of specific products and had a lower repressive effect on the genes. The same behavior presented by the yeast *P. pastoris* can be observed in the present work with *C. viswanathii*, which presented better results for enzymatic activity using sorbitol as substrate combined with vegetable oil. It was also verified that the largest biomass amounts were observed using residual glycerol as carbon source combined with vegetable oil.

Nonoleaginous carbon sources do not induce enzyme production, but positively help cell growth and consequently, enzyme synthesis. The use of combined carbon sources, simple carbohydrates and inducing sources allows an increase in enzyme production^26,30,32,33^ and, because of the high cell density, the final enzyme concentration in the medium is high.

Yeast extract was the best carbon source for enzyme production. This is a good source for lipase production and cell growth, since being organic, it has some additional nutritional factors that are necessary for cell development and yeast metabolism, such as vitamins, cofactors and amino acids.^34,35^ Erkmen and Fadiloğlu,^36^ while analyzing various organic nitrogen sources in submerged cultivation for lipase production by *Candida rugosa*, found that the highest enzyme production was observed with yeast extract and peptone (5.58 U mL^–1^). Regarding biomass production, yeast extract yielded the best results. Similarly, Pereira et al.^37^ used submerged cultivation with mango residues to evaluate lipase production by *Y. lipolytica*. After testing various organic and inorganic nitrogen sources, they concluded that yeast extract was the most promising.

The presence of cofactors in yeast extract may justify its preference by *C. viswanathii*. As observed by Erkmen and Fadiloğlu^36^ and Pereira et al.,^37^ organic nitrogen sources enhance enzyme yield. In addition to nitrogen being essential for cell development and metabolism, other components in yeast extract are also indispensable for cell growth. Although corncin did not show the best results for lipase production by *C. viswanathii*, it demonstrated reasonable enzyme and biomass production (Figure 1a,b). The concentration used in this study was 0.5% (w/v), and it is likely that a higher concentration could have yielded better performance. This assumption is supported by the findings of Maldonado et al.,^38^ who investigated submerged cultivation of *Geotrichum candidum* and observed that increasing the corncin concentration to 8% (w/v) (16 times higher than the concentration used in this study) resulted in an enzymatic yield of 21.0 U mL^–1^.

Among the nitrogen sources tested, yeast extract stood out as the most effective in terms of biomass and enzyme production, making it the chosen source for bioreactor analysis. Additionally, sorbitol and glycerol were selected as carbon sources for the screening of vegetable oils to assess their direct and indirect influence on lipase and biomass production, as they showed the highest values for enzyme and biomass production, respectively.

### 3.2 Selection of the Vegetable Oil

In this experiment it was evaluated the effect of several vegetable oils as a sole carbon source and with the supplementation of nonlipid carbon sources as adjunct for microbial growth and lipase production by *C. viswanathii* (Figure 2). It was observed that the supplementation of sorbitol in the cultures containing soybean oil, pequi oil or palm oil increased the lipase production in 68.4%, 62.7% and 38.0%, respectively (12.82 ± 0.7 U mL^–1^, 12.20 ± 0.7 U mL^–1^ and 9.42 ± 0.7 U mL^–1^, respectively) (Figure 2A). On the other hand, glycerol supplementation decreased the lipase production for all vegetable oil used in cultivations. Statistical analysis using Scott Knott test confirmed significant differences in lipase production among all experimental conditions (*p* > 0.05). When vegetable oil sources were evaluated as a sole carbon source and compared to olive oil it was observed that the use of sunflower oil induced the lipase production 45% higher than olive oil (10.21 ± 0.3 U mL^–1^). The use of palm oil + glycerol and pequi oil + glycerol resulted in the lowest lipase yields.

**Figure 2:**
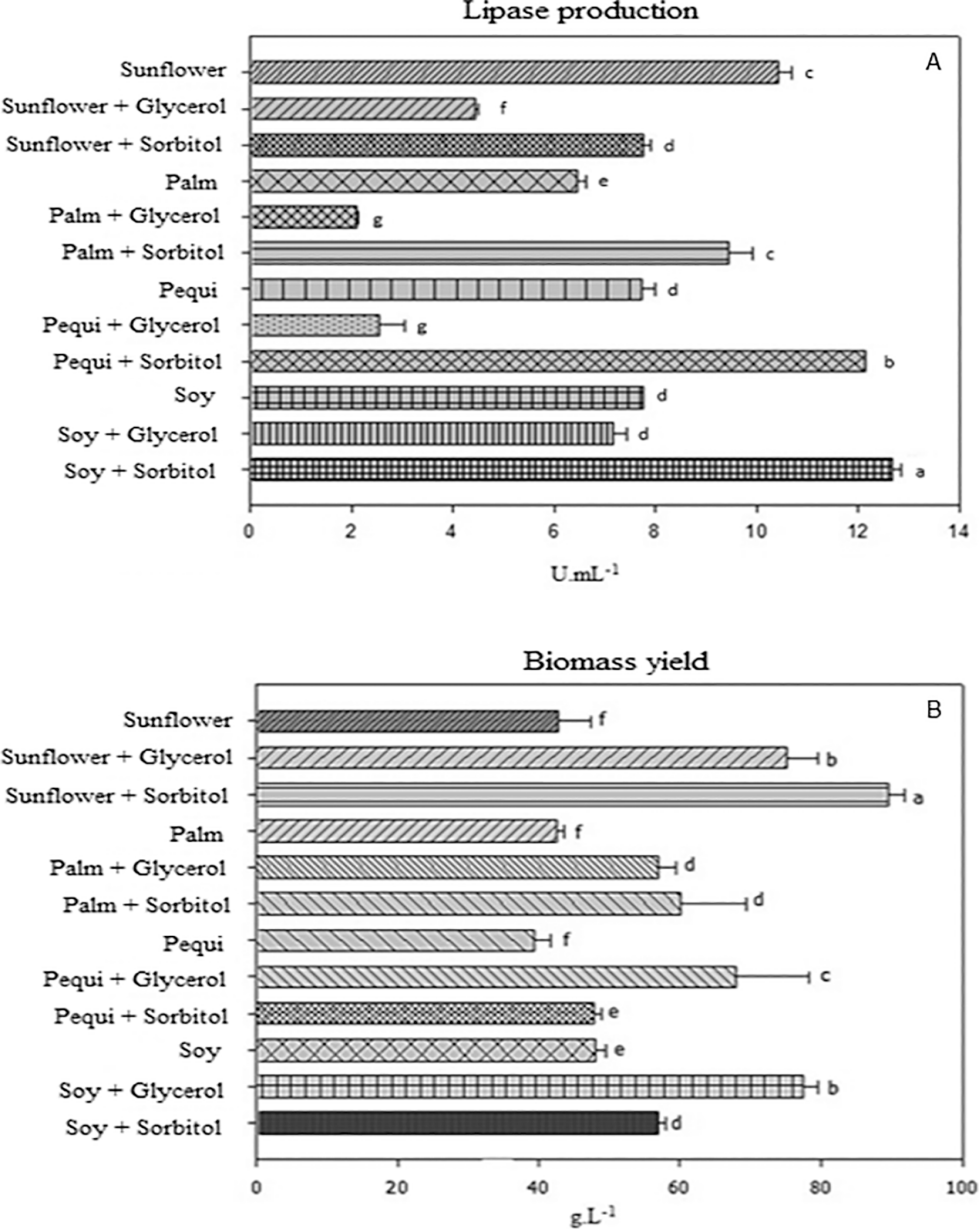
(A) enzymatic activity (U mL^–1^) and (B) biomass production (g·L^–1^). Distinct lowercase letters mean there are significant differences among the tested media at a 95% confidence interval.

For lipase production, the combinations of soybean oil + sorbitol and pequi oil + sorbitol differed statistically from each other. The palm oil + sorbitol and the medium with only sunflower oil as carbon source were similar at a 95% confidence interval. The cultivations with sunflower oil + sorbitol, pequi oil, soybean oil and soybean oil + glycerol were statistically similar. Palm oil + glycerol was like cultivation with pequi oil + glycerol, with the lowest yields. Differing from all crops, the combination sunflower oil + sorbitol presented the highest biomass production. From this result, it can be said that lipase production by *C. viswanathii* is not entirely associated with the increase in biomass, since the crop that presented the highest biomass was not the one that presented the best enzymatic activity.

Biomass production was significantly enhanced (*p* > 0.05) when the supplementation of the culture medium with nonlipid carbon sources (sorbitol and glycerol) was compared to cultivation with a sole vegetable oil (Figure 2B). The highest biomass production (89.0 g/L) was achieved with sunflower oil + sorbitol. Interestingly, the culture containing sunflower oil + sorbitol, which produced the highest biomass concentration, was statistically distinct from all other conditions, indicating that lipase production by *C. viswanathii* is not strictly correlated with biomass accumulation. In this study, residual glycerol inhibited the enzyme production when used as a supplementary carbon source. Glycerol can simultaneously enable microbial growth while reducing measurable extracellular lipase activity because metabolism and extracellular enzyme function are separable processes. Growth depends on intracellular glycerol catabolism, whereas extracellular lipase activity can be reduced by competitive binding or by physical blockage without preventing glycerol uptake.^39,40^ In other studies, glycerol is related as an inhibitor that promotes reduction in enzyme production when used as an adjunct in culture medium for enzyme production.^30,41,42^ This fact can be justified by the origin of the residual glycerol, which comes from the biodiesel chain, being a coproduct. Since glycerol is a residue from the transesterification of soybean oil in biodiesel, its composition may contain unconverted fatty acids, impurities such as alcohol and unrecovered catalysts, as well as a high concentration of lipids that can cause catabolic repression in enzyme metabolism.

de Almeida et al.^22^ observed the greatest growth and enzyme production in *C. viswanathii* with oleic acid and triolein as carbon sources at 2% (w/v) of medium supplementation. Using vegetable oils, the best results followed the order: olive oil, palm oil, canola oil, sunflower oil and soybean oil. These oils have in their composition long-chain fatty acids (C18) with and without unsaturation. The lipase from *C. viswanathii* is preferentially induced by the presence of unsaturated long-chain fatty acids.^11^ Oleic acid (C18:1) and linoleic acid (C18:2), present in soybean oil, are unsaturated long-chain fatty acids, a fact that may explain their higher enzyme yield. Fabiszewska et al.^30^ and Mohaini et al.^43^ screened carbon sources for lipase production and found positively influenced enzymatic activity using olive oil. Fabiszewska et al.^30^ related this behavior to the oleic acid present in olive oil.

### 3.3 Cultivation Conditions by RCCD Optimization

Table 2 presents the results of the rotational central compound design (RCCD). Based on these results, the highest lipase production is related to the highest amount of soybean oil (23.4 g·L^–1^) and the amount referring to the central points of yeast extract (7.5 g·L^–1^) and sorbitol (10 g·L^–1^), as sources of nitrogen and carbon, respectively. The requirement of sources that induce lipase biosynthesis is evident in several studies, and there may be variations in nitrogen concentrations and the presence or absence of other carbon sources.^13,26,41^ The presence of an oleaginous carbon source that induces microbial metabolism is essential for increases in enzyme yield, even for intracellular lipases and extracellular enzymes.^44^

**Table 2:** Responses Obtained with RCCD Used to Acquire Surface Responses with 3 Independent Variables for Lipase Production by *C. viswanathii* ^a^

experiments	vegetal oil (g·L^–1^)—*X*_1_	carbon source (g L^–1^)—*X*_2_	nitrogen source (g·L^–1^)—*X*_3_	enzymatic activity (U mL^–1^)—*Y*_1_
1	–1 (10)	–1 (5)	–1 (5)	5.14 ± 1.27
2	–1 (10)	–1 (5)	1 (10)	7.62 ± 1.74
3	–1 (10)	1 (15)	–1 (5)	7.55 ± 0.50
4	–1 (10)	1(15)	1(10)	7.44 ± 2.64
5	1 (20)	–1 (5)	–1 (5)	7.04 ± 0.81
6	1 (20)	–1 (5)	1 (10)	8.20 ± 1.67
7	1 (20)	1 (15)	–1 (5)	11.33 ± 0.25
8	1 (20)	1(15)	1 (10)	7.77 ± 0.22
9	–1.68 (6.6)	0 (10)	0 (7.5)	7.10 ± 0.88
10	1.68 (23.4)	0 (10)	0 (7.5)	13.56 ± 1.52
11	0 (15)	–1.68 (1.6)	0 (7.5)	4.13 ± 0.95
12	0 (15)	1.68 (18.4)	0 (7.5)	5.09 ± 0.29
13	0 (15)	0 (10)	–1.68 (3.3)	7.32 ± 0.57
14	0 (15)	0 (10)	1.68 (11.7)	7.72 ± 0.41
15C	0 (15)	0 (10)	0 (7.5)	8.26 ± 0.19
16C	0 (15)	0 (10)	0 (7.5)	9.03 ± 0.94
17C	0 (15)	0 (10)	0 (7.5)	8.42 ± 0.39

a The analysis of variance (ANOVA—Table 3) was presented for the quadratic model with interaction applied to the hydrolytic activity (*Y*_1_) of lipase production by *C. viswanathii*.

The adjustment of the experimental responses to the model was evaluated by the coefficient of determination (*R* ^2^) and the Fisher test (*F* test). The application of the *F* test to the model explains a significant amount of variation in the experimental data.^28^ The *R* ^2^ obtained indicates that 80.79% of the variability of the responses observed can be explained by the adjusted model (equation A). In biological processes, predictive models are considered adequate when the coefficient of determination (*R* ^2^) is equal to or greater than 80%, taking into account the inherent variability of biological systems and experimental conditions.^45^

1 $$Y_{1}\;\left(U/mL\right)=8.16+1.25\left[X_{1}\right]+0.78{\left[X_{1}\right]}^{2}-1.24{\left[X_{2}\right]}^{2}-0.95\left[X_{2}X_{3}\right]$$

where *X*_1_, *X*_2_ and *X*_3_ represent the levels of the factors vegetal oil, carbon and nitrogen sources, respectively.

In addition, by the *F* test, it was verified that the model explains a significant amount of variation of the experimental data, since for the level of significance of 5%, the calculated *F* value (12.6) was higher than tabulated *F* value (3.26) of a reference frequency distribution (*F*_degrees of the model; degrees of freedom of the residues; level of significance_).^46^ The *R* ^2^ and the *F* test results indicate that the variation explained by the model is greater than the unexplained residual variation, demonstrating that the model is statistically valid.

The response surfaces and contour curves (Figure 3), obtained from the fitted model (equation A), demonstrated that the highest hydrolytic activity values were achieved within an optimal region characterized by vegetable oil concentrations higher than 23 g/L, combined with carbon source concentrations ranging from 6 to 14 g/L (Figure 3A). High hydrolytic activity levels were also maintained over a broad range of nitrogen source concentrations, between 3 and 12 g/L (Figure 3B). In addition, the combination of nitrogen source concentrations in the range of 3–12 g/L with a narrower range of carbon source concentrations (9–11 g/L) favored the production of lipases from *C. viswanathii* exhibiting the highest hydrolytic activity values (Figure 3C), indicating a positive interaction between these variables within the evaluated experimental domain.

**Figure 3:**
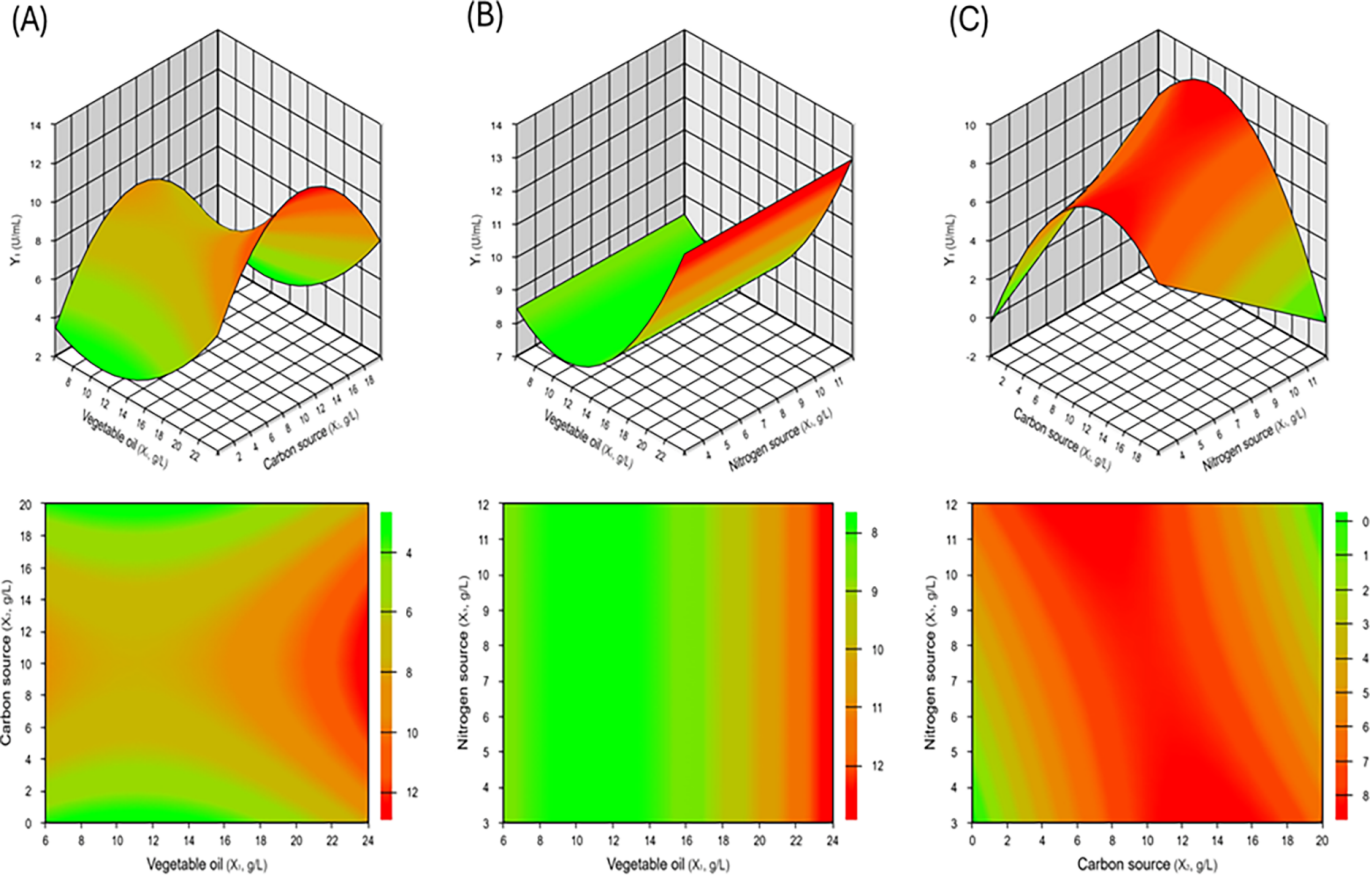
Response surface and contour curves showing the combined effects of (A) vegetable oil (g/L) and carbon source (g/L), (B) vegetable oil (g/L) and nitrogen source (g/L), and (C) carbon source (g/L) and nitrogen source (g/L) on the hydrolytic activity (U/mL).

Salihu et al.^47^ analyzed several parameters individually, using the Plackett–Burman experimental design. The authors observed that the nonlipid carbon source, glucose, had a significant negative effect on lipase production by *Candida cylindeacea*, and olive oil and the nitrogen source had a positive effect on this production. Salihu et al.^47^ observed that the interaction between the carbon source (tween 80) and the nitrogen source (peptone), using a central composite design, was significant for lipase production by *Candida cylindracea*. Kishan et al.,^48^ observed the effect of the nonlipid carbon source was not significant for lipase production by *Y. lipolytica*, and enzyme production was conditioned to higher concentrations of the oleaginous carbon source; however, the presence of a simple carbon source was necessary.

### 3.4 RDDC Validation

The maximum lipase production predicted by the experimental design was 13.56 ± 1.52 U mL^–1^, obtained with a medium composed of yeast extract, sorbitol and soybean oil at the concentrations of 7.5 g·L^–1^, 10 g·L^–1^ and 23.4 g·L^–1^, respectively. In these conditions, the experimental result obtained was 13.78 ± 0.23 U mL^–1^. Comparing the experimental results with the result predicted by RDDC, it is possible to state that this design was validated and the existence of an optimal production range for the tested conditions showed a considerable increase for the initial screenings (Table 3).

**Table 3:** Analysis of Variance (ANOVA) Results for the Quadratic Model with Interaction Used to Evaluate the Effects of Vegetable Oil Concentration and Carbon and Nitrogen Source Concentrations on the Hydrolytic Activity of Lipase from*C. viswanathii*, as Determined by RCCD (*p* ≤ 0.05)^a^

source of variation	sum of squares	degrees of freedom	mean square	*F*_calculated_	*p*-value
model	62.6	4	15.6	12.6	0.00029
residues	14.9	12	1.20		
lack of fit	14.5	10	1.50	8.8	0.10615
pure error	0.30	2	0.20		
total	77.5	16	-		

a *R* ^2^ = 80.79%. *F*_tabulated_ (4; 12; 0.05) = 3.26.

### 3.5 Cultivation in the Bench Bioreactor

Lipase production by *C. viswanathii* was investigated in a stirred tank bioreactor. The experiments were conducted in the absence and presence of sorbitol, a noninducer carbon source (Figure 4). Soybean oil consumption decreased from 23.4 g·L^–1^ to 7.5 g·L^–1^ after 4 h of cultivation (Figure 4A). The fast cell growth promoted this rate of oil consumption during the cultivation (Figure 4B). On the other hand, lipase production presented a lag phase of 8 h, reaching the highest lipase production after 32 h of cultivation (13.56 U mL^–1^) (Figure 4C). The metabolic adaptation of *C. viswanathii* was quicker, as it had the same composition as the inoculum used, facilitating both cell growth and lipase production. Elegado et al.^41^ observed that the microorganism’s adaptation phase in a bioreactor was reduced due to the similarity between the inoculum and the medium in which it was cultivated.

**Figure 4:**
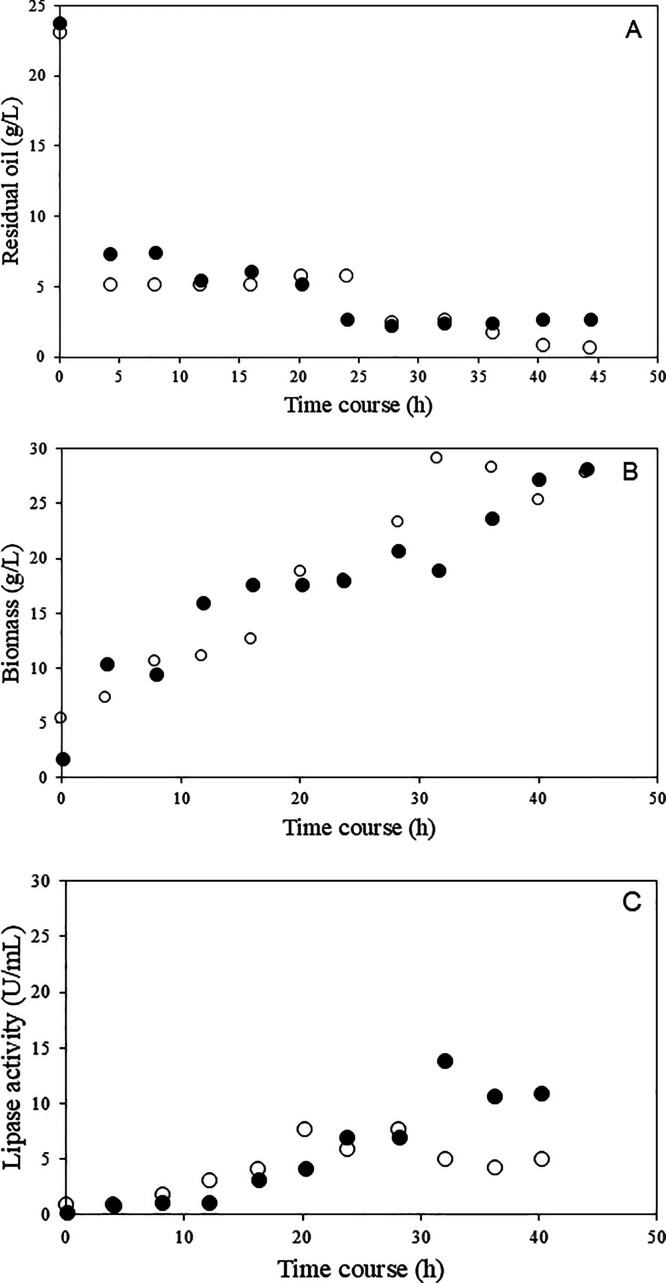
Cultivation in the stirred tank bioreactor with sorbitol (○) and in the absence of sorbitol (●). A: residual oil; B: biomass; and C: enzymatic activity.

The fluctuation of residual oil in the medium, observed between 12 and 20 h of cultivation, may be associated with the release of fatty acids that were not assimilated by the yeast. These compounds, once dispersed in the medium, were likely detected within the time frame of analysis. After 32 h of cultivation, a marked reduction in lipase activity was observed, reaching 10 U·mL^–1^. Such a decline can be explained by the inhibitory effect exerted by an excess of substrates, which is known to negatively regulate lipase biosynthesis. It is also plausible that this phenomenon occurred during the late stages of cultivation, when the accumulation of hydrolytic products could have promoted the degradation of lipases already secreted into the medium. This phenomenon was also reported by Imandi et al.^49^ and Elegado et al.^41^ These authors noted that hydrolysis of inducing lipid sources in the medium leads to the release of fatty acids. Excess substrates can cause lipase inhibition, thereby reducing its production. Additionally, yeasts from the *Candida* genus are known for their ability to synthesize proteases under stress conditions.^50–52^ This synthesis might have occurred at the end of the cultivation period, leading to the hydrolysis of lipases dispersed in the medium.

In the cultivation of *C. viswanathii* in the presence of sorbitol, vegetable oil consumption occurred later than in the cultivation without sorbitol. After 6 h of cultivation, the residual oil decreased from 23.4 g·L^–1^ to 5.5 g·L^–1^. The lag phase of enzyme production differed from that observed in the absence of sorbitol, with a significant increase in lipase production after 12 h of cultivation. However, lipase production was lower than in the absence of sorbitol, reaching only 6.69 U mL^–1^ after 32 h of cultivation. After this period, the enzyme concentration in the medium also began to decline, similar to the cultivation without sorbitol.

Cell growth significantly increased after the first 6 h of cultivation. The initial decay of residual oil in the medium coincided with the initial increase in biomass (after 6 h of cultivation). After this point, the oil concentration in the medium remained constant, and it is assumed that sorbitol consumption intensified, as cell growth continued at a notable rate. In contrast to the cultivation without sorbitol, biomass reached a higher value of 30 g·L^–1^ after 32 h, while the cultivation without sorbitol yielded only 25 g·L^–1^ in the same time. This clearly demonstrates the influence of sorbitol on biomass increase, since it functions as an easily assimilable carbon source, sorbitol provides additional carbon and energy to the cell during biosynthesis and maintenance metabolism, resulting in greater microbial growth.^53^ The presence of sorbitol in the cultures of *C. viswanathii* resulted in a 16.6% higher cell concentration compared to the cultivation conducted without it. Although the highest biomass concentration was observed in the medium, this factor can negatively impact enzyme yield. While low cell density is associated with reduced synthesis of specific products, excessively high cell densities may hinder product formation due to the accumulation of toxic residues or decreased dissolved oxygen levels, causing cellular stress.^41,50^

The addition of sorbitol to the STR cultures negatively affected enzyme yield, reducing production by 49.33% compared to the validation experiment in shake flasks. Elegado et al.^41^ investigated *Cryptococcus flavescens* in a stirred tank bioreactor and observed a 10-fold decrease in lipase yield after bench-scale optimization. In contrast, Salihu et al.,^38^ working with *C. cylindracea*, reported an increase in lipase production following optimization of agitation, aeration, and temperature parameters via response surface methodology. On the other hand, studies of recombinant lipase production in *P. pastoris* demonstrated that the most plausible explanation for the reduction in lipase production in sorbitol-based culture systems is a metabolic trade-off: the availability of an additional carbon source directed to cellular flow for biomass formation instead of recombinant protein synthesis and molecule production.^54,55^

Reduced enzyme production during scale-up from bench to stirred tank bioreactors has been widely reported for various enzymes and microorganisms.^41,56^ These findings highlight the need to optimize not only medium composition but also physical parameters in bioreactors, as they differ from those in bench-scale cultivation. Even when medium conditions remain unchanged, inherent process differences can negatively affect microbial growth and development.

Lipase production by *C. viswanathii* demonstrates superior biotechnological efficiency compared to other strains of the *Candida* genus described in the literature (Table 4), consolidating it as a robust cellular matrix for enzymatic secretion. Although the yield was slightly lower than that reported for *Candida guilliermondii* (18.0 U mL^–1^),^58^ both studies highlight soybean oil as a high-performance inducer in stirred-tank reactor (STR) systems. In contrast, the activity obtained by *C. viswanathii* significantly exceeded the values recorded for *C. rugosa* in triple-impeller bioreactors (1.84 U mL^–1^)^59^ and for the *Candida tropicalis* strains (URM 7057 ^60^ and ATCC750,^57^ respectively) cultivated in olive mill wastewater (OMW), whose activities ranged between 203 U L^–1^ and 2724 U L^–1^. This discrepancy of orders of magnitude underscores that, while the use of agro-industrial residues such as OMW is environmentally advantageous, *C. viswanathii* exhibits metabolic plasticity and a secretion rate in media supplemented with vegetable oils that strategically position it for industrial processes requiring high enzymatic concentrations in smaller cultivation volumes.

**Table 4:** Lipase Production by *Candida* Genus in Bioreactor

Candia genus	cultivations system	main component culture medium	lipase production	references
C. viswanathii	STR cultivation	soybean oil; yeast extract	13.65 U mL^–1^	this work
C. tropicalis ATCC 750	STR cultivation	olive mill wastewater	203 U L^–1^	Dias et al.^57^
C. guilliermondii	batch fermentation	soybean oil	18.0 U mL^–1^	Oliveira et al.^58^
C. rugosa	STR triple impeller	olive oil	1.84 U mL^–1^	Puthli et al.^59^
C. tropicalis URM 7057	STR cultivation	olive mill wastewater	2724 U L^–1^	Silva^60^

Biomass yield and enzyme production per substrate consumed, biomass and enzyme productivity per hour of cultivation, and the specific rate of lipase production were obtained (Figures 5 and 6). Biomass yield per substrate consumed (*Y*_X/S_) (Figure 5A), in the cultivation with sorbitol, presented higher results after 44 h of cultivation (2.03 w/w). The highest biomass yield per substrate consumed for cultivation in the absence of sorbitol was 1.60 w/w in 40 h of cultivation. This result confirms that the presence of sorbitol in the culture medium is related to cell growth, in this case, of the yeast *C. viswanathii*. The highest yield for lipase yield per substrate consumed (Figure 5B), is related to the cultivation conducted in the absence of sorbitol. A significant increase occurs between 28 and 32 h of cultivation (1.24 U g^–1^ to 3.22 U g^–1^). In the presence of sorbitol, the maximum yield is observed after 24 h of cultivation (1.80 U g^–1^), with a decrease throughout cultivation, remaining below what was observed in the absence of sorbitol. de Almeida et al.,^22^ verifying different cultivations speeds of *C. viswanathii*, the best yields of *Y*_X/S_ and *Y*_L/S_ were observed after 72 h of cultivation, using olive oil as substrate, achieving the values 1.45 w/w and 6.63 U g^–1^, respectively.

**Figure 5:**
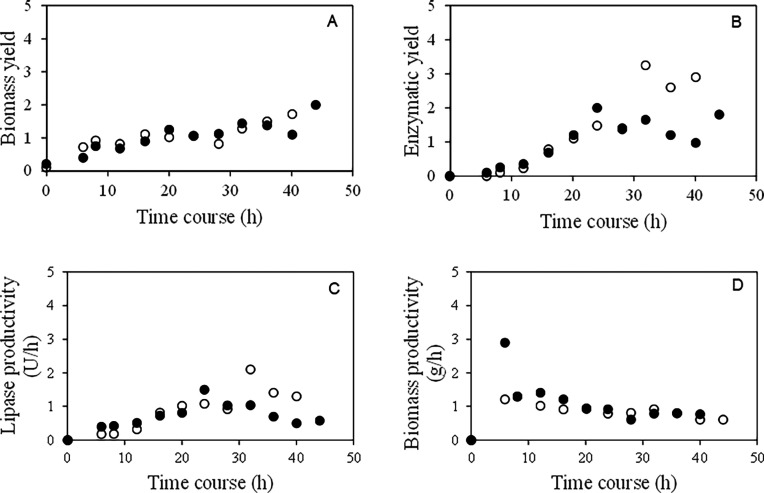
(A) Biomass yield per substrate consumed, (B) enzyme yield per substrate consumed, (C) lipase productivity per hour and (D) biomass productivity per hour, for the cultivation in a stirred tank bioreactor in (●) the presence of sorbitol and in (○) the absence of sorbitol for lipase production by *C. viswanathii*.

**Figure 6:**
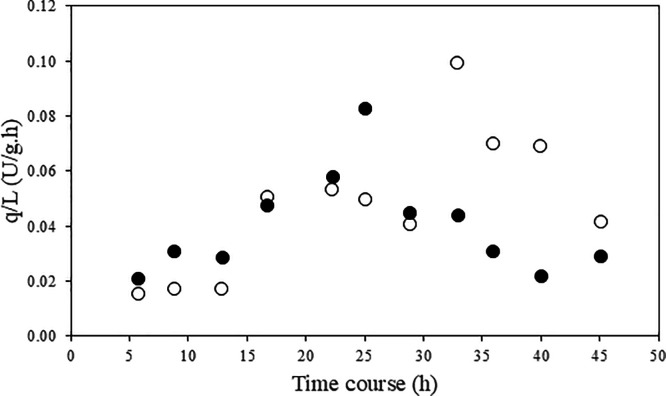
Specific rate of lipase production in a culture in a stirred tank bioreactor in (●) the presence of sorbitol and in (○) the absence of sorbitol for lipase production by *C. viswanathii*.

For the productivity per hour of cultivation, lipase productivity (Figure 5c) was higher in the cultivation performed in the absence of sorbitol (*P*_L_ 2.05 U h^–1^ after 32 h of cultivation). In the cultivation conducted with the addition of sorbitol to the medium, there was a trend of productivity decrease after 32 h of cultivation (*P*_L_ of 32 h of cultivation from 1.04 U h^–1^ to 0.71 U h^–1^ in 36 h of cultivation). For biomass productivity (Figure 5D), in the initial hours, the highest productivity was observed for the cultivation in the absence of sorbitol, with a *P*_*x*_ of 2.87 g h^–1^ in 4 h of cultivation, decreasing thereafter. Comparing the two cultures in relation to biomass productivity, after 12 h of fermentation, similar productivity behaviors were observed. The specific rate of lipase production (*q*_L_) (Figure 6) at 32 h and in the absence of sorbitol was 0.1 U/g/h. With sorbitol in the medium, the highest *q*_L_ was observed at 24 h of cultivation, being 0.08 U/g/h. de Almeida et al.^20^ worked with *C. viswanathii* and used only vegetable oil as carbon source and obtained a specific rate (*q*_L_) of 0.064 U/g/h after 72 h of cultivation. This value is lower than that obtained after 32 h of cultivation in the absence of sorbitol (0.1 U/g/h) and at 24 h of culture in the presence of sorbitol (0.08 U/g h). Teixeira^61^ worked with *C. viswanathii* aiming at lipase production. When testing the supplementation of soybean oil at 2% in the medium as a substrate, yields of *P*_*x*_ 1.01 g/g, *P*_L_ 3.63 and *q*_L_ of 0.005 were obtained. The productivity observed in the work of Teixeira^61^ were higher than those obtained in the present study; nevertheless, the specific rate, in this work, of lipase production in the culture without sorbitol was higher than that observed by those authors.

## 4 Conclusion

Overall, the findings of this study indicate that the metabolic efficiency of *C. viswanathii* for lipase production is intrinsically linked to the synergy between carbon source composition and the physical dynamics of the cultivation system. While oils and yeast extract remain fundamental for induction, the presence of sorbitol emerged as a critical regulatory factor, exerting an inhibitory effect on enzyme expression regardless of the scale. Most importantly, the transition from shaken flasks to stirred tank bioreactors reveals that the yeast’s physiological response is highly sensitive to oxygen transfer and mixing conditions. These findings suggest that for the industrial scale-up of *C. viswanathii* lipases, the optimization must transcend nutritional requirements, prioritizing the control of aeration and agitation as primary drivers of enzymatic induction and biomass yield.

## References

[1] Kumar A., Dhiman S., Krishan B., Samtiya M., Kumari A., Pathak N., Kumari A., Aluko R. E., Dhewa T. (2024). Microbial
enzymes and major applications in the food industry: a concise review. Food Prod., Process. Nutr..

[2] Farhan M., Hasani I. W., Khafaga D. S. R., Ragab W. M., Ahmed
Kazi R. N., Aatif M., Muteeb G., Fahim Y. A. (2025). Enzymes
as catalysts in industrial biocatalysis: advances in engineering,
applications, and sustainable integration. Catalysts.

[3] Fortune Business Insight . Lipase Market Size, Share & Industry Analysis, By Source (Microbial and Animal), By Application (Food & Beverage {Bakery, Dairy, Confectionery, Beverages and Others}, Nutraceuticals, Animal Feed, and Others), and Regional Forecast, 2026–2034, 2026. https://www.fortunebusinessinsights.com/lipase-market-110345 (accessed Feb 13, 2026).

[4] dos
Santos L. N., Perna R. F., Vieira A. C., de Almeida A. F., Ferreira N. R. (2023). Trends in the use of lipases: a systematic review and
bibliometric analysis. Foods.

[5] Salgado C. A., Santos C. I. A., Vanetti M. C. D. (2022). Microbial lipases: propitious biocatalysts
for the food industry. Food Biosc..

[6] Peng Q., Wang X., Shang M., Huang J., Guan G., Li Y., Shi B. (2014). Isolation of a novel
alkaline-stable lipase from a
metagenomic library and its specific application for milkfat flavor
production. Microb. Cell Fact..

[7] Meneses A. C., Almeida S. A. G., Lerin L. A., Corazza M. L. (2019). Benzyl
butyrate esterification mediated by immobilized lipases: Evaluation
of batch and fed-batch reactors to overcome lipase-acid deactivation. Process Biochem..

[8] Francolini I., Taresco V., Martinelli A., Piozzi A. (2020). Enhanced performance
of Candida rugosa lipase immobilized onto alkyl chain modified-magnetic
nanocomposites. Enzyme Microb. Technol..

[9] Tarafdar A., Sirohi R., Gaur V. K., Kumar S., Sharma P., Varjani S., Pandey H. O., Sindhu R., Madhavan A., Rajasekharan R., Sim S. J. (2021). Engineering interventions in enzyme
production: lab to industrial scale. Biores.
Technol..

[10] Colla L. M., Primaz A. L., Benedetti S., Loss R. A. (2016). Surface
response methodology for the optimization of lipase production under
submerged fermentation by filamentous fungi. Braz. J. Microbiol..

[11] Lima L. G. R., Gonçalves M. M., Couri S., Melo V. F., Sant’Ana G. C. F., Costa A. C. A. d. (2019). Lipase production
by Aspergillus niger C by submerged fermentation. Braz. Arch. Biol. Technol..

[12] Wong L. Y., Saad W. Z., Mohamad R., Tahir P. M. (2017). Optimization of
cultural conditions for polygalacturonase production by a newly isolated
Aspergillus fumigatus R6 capable of retting kenaf. Ind. Crops Prod..

[13] Colla L. M., Primaz A. L., Benedetti S., Loss R. A. (2016). Surface
response methodology for the optimization of lipase production under
submerged fermentation by filamentous fungi. Braz. J. Microbiol..

[14] Lau H. L., Wong F. W. F., Rahman R. N. Z. R. A., Mohamed M. S., Ariff A., Hii S. L. (2023). Optimization of
fermentation medium components by response
surface methodology (RSM) and artificial neural network hybrid with
genetic algorithm (ANN-GA) for lipase production by Burkholderia cenocepacia
ST8 using used automotive engine oil as substrate. Biocatal. Agric. Biotechnol..

[15] Dar M. A., Loedji M. A. C., Lunggani A. T., Napitupulu T. P., Kanti A. I., Sudiana M. (2025). Statistical optimization
of culture
media components for enhanced production of lipase by lipolytic yeasts,
Pichia sp. and Trichosporon coremiiforme using response surface methodology. Biomass Conv. Bioref..

[16] S S., Chellapandi P. (2025). Process parameter
optimization for scalable extracellular
lipase production by Bacillus isolates under submerged fermentation. Bioresour. Technol. Rep..

[17] Lima L. G. R., Gonçalves M. M., Couri S., Melo V. F., Sant’Ana G. C. F., Costa A. C. A. d. (2019). Lipase production
by Aspergillus niger C by submerged fermentation. Braz. Arch. Biol. Technol..

[18] Maceno M. A. C., de Souza Vandenberghe L. P., Woiciechowski A. L., Soccol C. R., Spier M. R. (2016). Production of cellulases by Phanerochaete
sp. using empty fruit bunches of palm (EFB) as substrate: optimization
and scale-up of process in bubble column and stirred tank bioreactors
(STR). Waste Biomass Valor..

[19] Mello A. F. M., Souza Vandenberghe L. P., Herrmann L. W., Letti L. A. J., Burgos W. J. M., Scapini T., Manzoki M. C., Oliveira P. Z., Soccol C. R. (2024). Strategies and engineering
aspects on the scale-up
of bioreactors for different bioprocesses. Syst.
Microbiol. Biomanuf..

[20] de
Almeida A. F., Taulk-Tornisielo S. M., Carmona E. C. (2013). Influence of carbon
and nitrogen sources on lipase production by a newly isolated Candida
viswanathii strain. Ann. Microbiol..

[21] Gomes N. B., Dias K. B., Teixeira M. F. N., Santos C. C. A. A., Almeida A. F. (2018). Medium composition
and Amazonian oils for lipase production
by Candida viswanathii. Acta Scienti. Technol..

[22] de
Almeida A. F., Tauk-Tornisielo S. M., Carmona E. C. (2013). Acid Lipase from
Candida viswanathii: production, biochemical properties, and potential
application. BioMed Res. Int..

[23] Dias K. B., Oliveira N. M. L., Brasil B. S. A. F., Vieira-Almeida E. C., Paula-Elias F. C., Almeida A. F. (2021). Simultaneous high nutritional single
cell oil and lipase production by Candida viswanathii. Acta Sci. Polym. Technol. Aliment..

[24] de
Souza M. R., Teixeira R. C., Daúde M. M., Augusto A. N. L., Ságio S. A., Almeida A. F., Barreto H. G. (2021). Comparative
assessment of three RNA extraction methods for obtaining high-quality
RNA from Candida viswanathii biomass. J. Microbiol.
Methods.

[25] Daúde M. M., Teixeira R. C., Cardon C. H., de Araujo Santos G. C., de Almeida A. F., Chalfun-Junior A., Barreto H. G. (2023). Selection and validation
of reference genes for RT-qPCR gene expression studies in Candida
viswanathii cultivated under different grown conditions. J. Microbiol. Methods.

[26] Dalmau E., Montesinos J. L., Lotti M., Casas C. (2000). Effect of different
carbon sources on lipase production by Candida rugosa. Enzyme Microb. Technol..

[27] Almeida A. F., Terrasan C. R. F., Terrone C. C., Tauk-Tornisielo S. M., Carmona E. C. (2018). Biochemical properties of free and immobilized Candida
viswanathii lipase on octyl-agarose support: Hydrolysis of triacylglycerol
and soy lecithin. Process Biochem..

[28] Rodrigues, M. I. ; Costa, P. Protimiza Experimental Design, version 1; Protimiza, Campinas, Brazil, 2014. https://experimental-design.protimiza.com.br/ (accessed Feb 17, 2026).

[29] Kumar A., Verma V., Dubey V. K., Srivastava A., Garg S. K., Singh V. P., Arora P. K. (2023). Industrial
applications
of fungal lipases: a review. Front. Microbiol..

[30] Fabiszewska A. U., Kotyrba D., Nowak D. (2015). Assortment of carbon
sources in medium
for Yarrowia lipolytica lipase production: A statistical approach. Ann. Microbiol..

[31] Thorpe E. D., D’Anjou M. C., Daugulis A. J. (1999). Sorbitol as a non-repressing
carbon
source for fed-batch fermentation of recombinant Pichia pastoris. Biotechnol. Lett..

[32] Muralidhar R. V., Chirumamila R. R., Marchant R., Nigam P. (2001). A response surface
approach for the comparison of lipase production by Candida cylindracea
using two different carbon sources. Biochem.
Eng. J..

[33] Galvagno M. A., Iannone L. J., Bianchi J., Kronberg F. (2011). Optimization
of biomass production of a mutant of Yarrowia lipolytica with an increased
lipase activity using raw glycerol. Rev. Argent.
Microbiol..

[34] Tan T., Zhang M., Wang B., Ying C., Deng L. (2003). Screening
of high lipase producing Candida sp. and production of lipase by fermentation. Process Biochem..

[35] Liu Y., Zhang Y. G., Zhang R. B., Zhang F., Zhu J. (2011). Glycerol/Glucose
co-fermentation: one more proficient process to produce propionic
acid by Propionibacterium acidipropionici. Curr.
Microbiol..

[36] Fadiloğlu S., Erkmen O. (2002). Effects of carbon and
nitrogen sources on lipase production
by Candida rugosa. Turkish J. Eng. Environ.
Sci..

[37] Pereira A. d. S., Fontes-Sant’Ana G. C., Amaral P. F. F. (2019). Mango agro-industrial
wastes for lipase production from Yarrowia lipolytica and the potential
of the fermented solid as a biocatalyst. Food
Bioprod. Process..

[38] Maldonado R. R., Aguiar-Oliveira E., Pozza E. L., Costa F. A. A., Filho F. M., Rodrigues M. I. (2014). Production
of lipase from Geotrichum candidum using
corn steep liquor in different bioreactors. JAOCS.

[39] Tian M., Wang Z. Y., Fu J. Y., Li H. W., Zhang J., Zhang X. F., Luo W., Lv P. M. (2021). Crude glycerol impurities
improve Rhizomucor miehei lipase production by Pichia pastoris. Prep. Biochem. Biotechnol..

[40] Serpa J. D. M., Guimarães N. C.
A., Yonekawa M. A. K., Almeida A. P., Ruller R., Jaques J. A. S., Santos E. A., Masui D. C., Zanoelo F. F., Giannesi G. C. (2022). Sarocladium strictum
lipase (LipSs)
produced using crude glycerol as sole carbon source: A promising enzyme
for biodiesel production. Biocatal. Agric. Biotechnol..

[41] Elegado F., Legaspi C. L., Paet J. M., Querubin F., Tolentino J. E., Vilela J., Paguio A., Maloles J., Zarate J. (2019). Screening,
identification and optimization of extracellular lipase production
of yeast (Cryptococcus flavescens) isolated from a tree canopy fern
in the Mount Makiling Forest Reserve, Philippines. AIP Conf. Proc..

[42] Robert J.
M., Lattari F. S., Machado A. C., de Castro A. M. (2017). Production of recombinant
lipase B from Candida antarctica in Pichia
pastoris under control of the promoter PGK using crude glycerol from
biodiesel production as carbon source. Biochem.
Eng. J..

[43] Mohaini M. A., Farid A., Muzammal M., Ghazanfar S. (2022). Enhancing lipase production of Bacillus salmalaya
strain 139si using
different carbon sources and surfactants. Appl.
Environ. Microbiol..

[44] Makhsumkhanov A. A., Yakubov I. T., Davranov K. (2003). Conditions for cultivation
of the
fungus Penicillium melinii UzLM-4 and its biosynthesis of lipases. Appl. Biochem. Microbiol..

[45] Pereira R. S., Vieira A. C., Leite P. C., Maestrelli S. C., Silva E. S., Maiorano A. E., Xavier M. C. A., Lopes M. S., Paula A. V., Morales S. A. V., Perna R. F. (2025). Application of an
agro-waste for the immobilization of microbial fructosyltransferase:
a new alternative for fructooligosaccharide production. J. Braz. Chem. Soc..

[46] Garcia R. L., Dias G. S., Morales S. A. V., Xavier M. C. A., Silva E. S., Maiorano A. E., Tardioli P. W., Perna R. F. (2021). Glutaraldehyde-crosslinked
cells from Aspergillus oryzae IPT-301 for high transfructosylation
activity: optimization of the immobilization variables, characterization
and operational stability. Braz. J. Chem. Eng..

[47] Salihu A., Alam M. Z., Abdulkarim M. I., Salleh H. M. (2011). Effect of process
parameters on lipase production by Candida cylindracea in stirred
tank bioreactor using renewable palm oil mill effluent based medium. J. Mol. Catal. B:Enzym..

[48] Kishan G., Gopalakannan P., Muthukumaran C., Thirumalai Muthukumaresan K., Dharmendira Kumar M., Tamilarasan K. (2013). Statistical optimization of critical
medium components for lipase production from Yarrowia lipolytica (MTCC
35). J. Gen. Eng. Biotechnol..

[49] Imandi S. B., Karanam S. K., Garapati H. R. (2013). Use of
Plackett-Burman design for
rapid screening of nitrogen and carbon sources for the production
of lipase in solid state fermentation by Yarrowia lipolytica from
mustard oil cake (Brassica napus). Braz. J.
Microbiol..

[50] Gropp K., Schild L., Schindler S., Hube B., Zipfel P. F., Skerka C. (2009). The yeast Candida albicans
evades human complement
attack by secretion of aspartic proteases. Mol.
Immunol..

[51] Dutton L. C., Jenkinson H. F., Lamont R. J., Nobbs A. H. (2016). Role of Candida
albicans secreted aspartyl protease Sap9 in interkingdom biofilm formation. Pathog. Dis..

[52] Rasheed M., Battu A., Kaur R. (2018). Aspartyl proteases
in Candida glabrata
are required for suppression of the host innate immune response. J. Biol. Chem..

[53] Arnau C., Ramon R., Casas C., Valero F. (2010). Optimization of the
heterologous production of a Rhizopus oryzae lipase in Pichia pastoris
system using mixed substrates on controlled fed-batch bioprocess. Enzym. Microb. Technol..

[54] Velastegui E., Quezada J., Altamirano C., Berrios J., Fickers P. (2023). Co-feeding
strategy alleviates hypoxic stress in large-scale bioreactor for optimal
production of secretory recombinant proteins in Pichia pastoris. J. Biotechnol..

[55] Vieira E. D., Andrietta M. d. G. S., Andrietta S. R. (2013). Yeast biomass production: a new approach
in glucose-limited feeding strategy. Braz. J.
Microbiol..

[56] Deive F. J., Sanromán M. A., Longo M. A. (2010). A comprehensive study of lipase production
by Yarrowia lipolytica CECT 1240 (ATCC 18942): from shake flask to
continuous bioreactor. J. Chem. Technol. Biotechnol..

[57] Dias B., Lopes M., Ramôa R., Pereira A. S., Belo I. (2021). Candida tropicalis
as a promising oleaginous yeast for olive mill wastewater bioconversion. Energies.

[58] Oliveira A. C. D., Fernandes M. L., Mariano A. B. (2014). Production and characterization of
an extracellular lipase from Candida guilliermondii. Braz. J. Microbiol..

[59] Puthli M. S., Rathod V. K., Pandit A. B. (2006). Optimization of lipase production
in a triple impeller bioreactor. Biochem. Eng.
J..

[60] Silva, R. K. P. Produção de lipase por Candida tropicalis URM 7057 utilizando biorreator tanque agitado, Dissertation, Federal University of Ceará, 2019. Available: https://repositorio.ufc.br/handle/riufc/41479 (accessed Feb 15 2026).

[61] Teixeira, N. F. N. Produção de lipase por Candida viswanathii: otimização das condições de cultivo, purificação em sistema aquoso bifásico e propriedades bioquímicas; Dissertation, Federal University of Tocantins, 2017.

